# Allowing family visits during COVID‐19 pandemic: A family‐centred moderate restrictive visitation programme in an intensive care unit

**DOI:** 10.1002/nop2.1254

**Published:** 2022-05-23

**Authors:** Shulan Yang, Huijuan Zhang, Fang Chen, Caixia Liu

**Affiliations:** ^1^ Nursing Department Zhejiang Hospital Hangzhou China; ^2^ Department of Critical Care Medicine Zhejiang Hospital Hangzhou China

## 1 BACKGROUND

The pandemic of COVID‐19 has caused much concern and changes in intensive care unit (ICU), including the sudden interruption of “opening” ICU (Mistraletti et al., 2021). During the pandemic, family members are prohibited from visitation based on the physical isolation policy. The impossibility of routine family visits poses a challenge for healthcare professionals, especially those in ICU.

In China, many ICUs have adopted WeChat to facilitate remote virtual family visits by means of instant text messaging and video calling. Worldwide, phone and video callings are widely used in communicating with families in the context of physical isolation (Mistraletti et al., 2020). The advantage of remote virtual visitation is preventing people from entering the patient unit, which lowers the risk of patients' exposure to COVID‐19. But it is difficult for the family to fully communicate with the care team via video calls or messages when it comes to important medical decisions. Actually, family‐clinician shared decision‐making significantly improved families' satisfaction and depression, shortened patients' duration of ICU stay, and enhanced ICU clinicians' collaboration (Liu et al., 2021). Family plays an essential role in decision‐making in ICU. Family support in times of the COVID‐19 crisis is important, as the family–patient communication is restricted and the family has a strong need for information and support (Klop et al., 2021).

Different from above, we constructed a family‐centred moderate restrictive visitation programme, which might be a good choice applied during the pandemic of droplet transmitted infection disease such as COVID‐19. For critically ill patients, virtual visitation can hardly meet the care and emotional needs of their families. Therefore, when constructing the family‐centred moderate restrictive visitation programme, visitors are stopped from accessing the ward, but allowed to enter the hospital in‐person, in collaboration with the updated hospital visitation policies. The in‐person visitation is limited to the greatest extent possible considering the risk of COVID‐19 transmission.

## 2 COVID‐19 TRANSMISSION PREVENTION MEASURES

In the proposed family‐centred moderate restrictive visitation programme, face‐to‐face communication with the ICU team is supported. Physical environmental protections are strengthened as we set up a separate communication room outside ICU. In the meanwhile, COVID‐19 transmission prevention measures are implemented prior to the visits, especially screening for epidemic exposure. In China, a colour‐based “health code” system has been widely adopted to detect the possible exposures to Covid‐19 since the crisis, relying on mobile technology and big data facilitated contact tracing.In the “health code” system, colours of the QR codes indicate people’s risk of Covid‐19 exposures. People with green, amber or red coloured code indicate low, moderate or high risk of epidemic exposure to Covid‐19 respectively. Nowadays in China, the “health code” system is widely applied in the health systems. The green coloured health code is considered as a necessary condition of accessing the routine health services, in case of lowering the risk of Covid‐19 transmissions. Patients with amber codes or red codes are placed in separated areas different from the low‐risk patient, such as fever clinic or Isolation ward. No access to visitors without green codes. Please refer to the figure of a diagram of the Family‐centred Moderate Restrictive Visitation Program in the separate file, in which the demo of the “health code” system is presented by illustration.
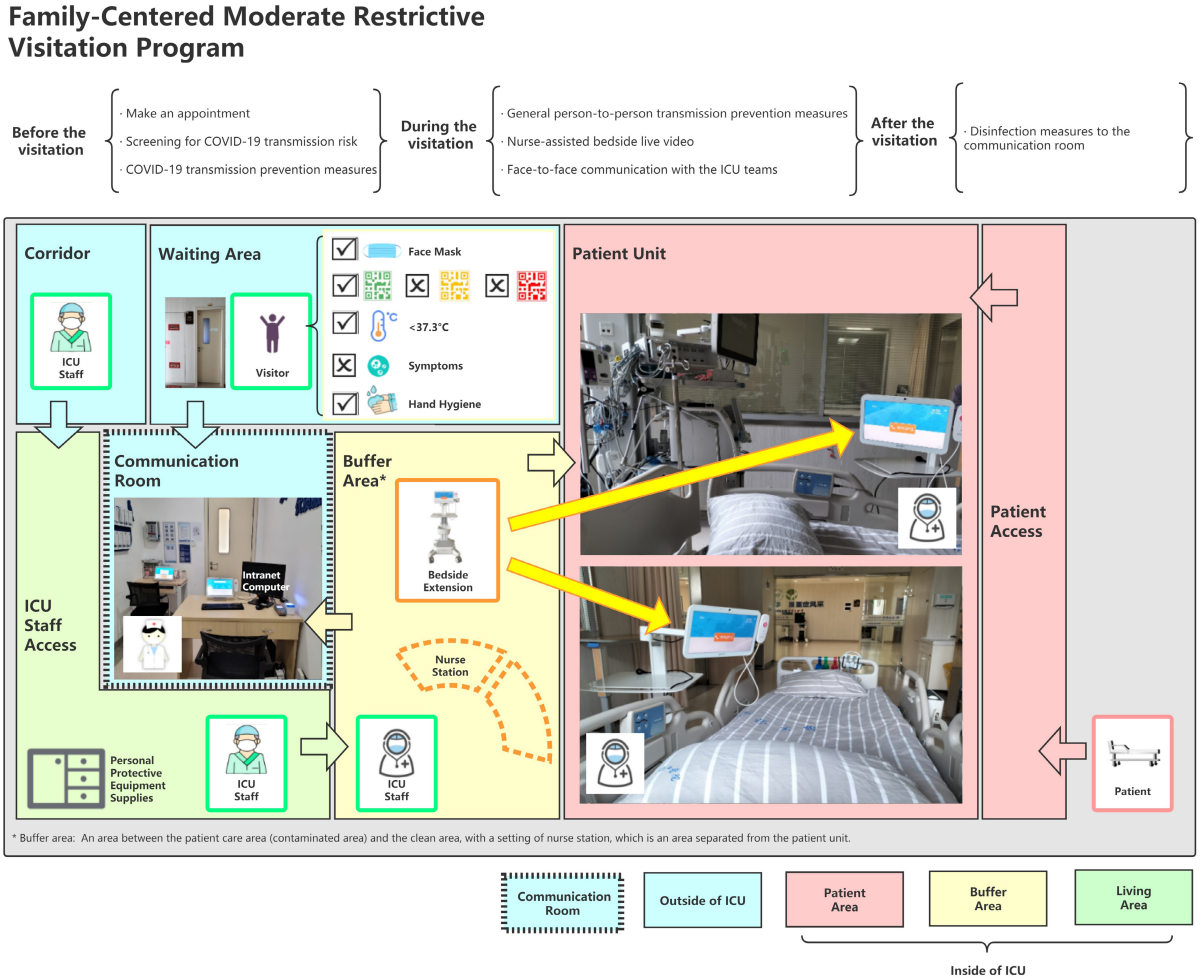


All visitors are screened for symptoms, temperatures and green codes. Any individuals with symptoms or suspicious exposures are stopped from entering the routine outpatient or inpatient service and are appointed to a certain area, such as fever clinics. All individuals in the inpatient area are tested for SARS‐CoV‐2 and are negative. Based on the above, we required all family visitors of ICU to provide their own negative SARS‐CoV‐2 report, to apply for a local authorized green‐coloured health code, and to receive screening for symptoms, temperatures when entering the hospital and before entering the communication room. Only individuals who are negative for SARS‐CoV‐2 have green codes, and are free of symptoms are allowed to access the communication room. Generally, the health code system is a simple digitalized method to detect epidemic exposure. Using the health code system combined with SARS‐CoV‐2 test and symptom status can efficiently prevent visitors of high‐risk Covid‐19 exposures from entering the hospital.

During the visitation, some general person‐to‐person transmission prevention measures are implemented, such as wearing face‐masks, performing hand hygiene, maintaining appropriate social distance etc (Chu et al., 2020). Since visitors do not need to enter the ICU, extra personal protective equipment (PPE) for visitors, such as a gown or overall, is not required. Visitors are only required to wear face‐masks, the same requirement as in public areas in the hospital. It avoids visitor’s risk of infection caused by inappropriate donning and doffing and reduces the use of PPE. And after the visitation, disinfection measures are performed to the whole room.

## 3 FAMILY‐CENTRED MODERATE RESTRICTIVE VISITATION

In the family‐centred moderate restrictive visitation programme, family‐centred is reflected in family‐led appointment and participation in important medical decisions. Firstly, we have set up a separated communication room outside the ICU, equipped with an intranet computer with the Hospital Information System and a large‐screen video phone connected to the bedside extension, a wireless handcar videophone, via intranet as well. Secondly, a simple visitation appointment system is built on an online survey tool. Their family members are able to make appointments in advance online. And the everyday appointments are scheduled automatically. No additional human resource is required in arranging the appointments. In the meanwhile, the visitation limits of “no more than one visitor per patient at a time” are achieved by the online appointment system. Reservations are open to all families with visiting needs from 13:30 to 17:00 every day. The visiting time is limited to <30 minutes for each family.

During the visitation, the appointed families are invited separately to stay in the communication room accompanied by the charge nurse on duty. The charge nurse is required to re‐assess the health codes, symptoms and SARS‐CoV‐2 test results. The position of the charge nurse during visitation is also needed before the pandemic, but the content of the responsibility is altered. In the 30‐minute time, the charge nurse would briefly introduce the condition and treatment progress of the patient and let the family communicate with the patient via live video calls. The video calls are done using a secure system via the hospital intranet, and the leakage of the patient’s private information is somehow prevented. The charge nurse would also explain to the family when they were confused with the information they received from the video calls. In many cases, family members of ICU patient, usually the ones authorized, are involved in medical decision‐making. In the family‐centred moderate restrictive programme, families are allowed to enter the communication room in the hospital and the face‐to‐face communication between family and ICU team is preserved. The ICU team is able to use all accessible patient information in the intranet computer to let the family fully understand the treatment progress, and to assist the family with informed consent. Please find the figure of a diagram of the Family‐centred Moderate Restrictive Visitation Program in the separate file, in which the programme is introduced in depth by illustration.

Behind the video call, the members of the whole ICU team are involved in the programme. When the charge nurse keeps the family accompanied in the visitation room during the video call, the care team would cope with the patient by bedside as needed. And in special cases, the patient’s doctor in charge would communicate with the family in the visitation room. All members are part of the programme, help to build the communication bridge between critically ill patients and their families, and deliver the family‐centred care.

Overall in the Family‐centred Moderate Restrictive Visitation Program, both in‐person and virtual visitation are included. Family visits are opened to the greatest extent in the acceptable limits under the corona‐virus crisis. In a 19‐month‐time practice since 2020, we gradually obtained the understanding of the patients and their families. In cases of some low‐compliance patients, the family even became part of the ICU team in persuading the patient to better cooperate with the care team. In our experience, no matter what kind of techniques are used, the ICU team is always the best communication bridge between critically ill patients and their families. And in the proposed programme, the ICU team supports both the family and the patient from both sides of the video calls, helping the family understand the situation of the patient and the patient coping with their treatment.

## 4 CONCLUSION

The proposed Family‐centred Moderate Restrictive Visitation Program in this study broadens the boundaries of family communication in ICUs in the context of COVID‐19 pandemic, which gives a new thought on adapting the nursing management to the Post‐Covid “new normal.” The new normal Covid‐19 is here to stay, and the world cannot be isolated forever. Post‐Covid “new normal” is on its way to come. New rules in nursing management are going to be considered under the concern of the normal COVID‐19 pandemic.

## AUTHOR CONTRIBUTIONS

Shulan Yang and Huijuan Zhang were the major contributors in writing and revising the manuscript. All authors read and approved the final manuscript.

## CONFLICT OF INTEREST

The authors declare that they have no competing interests.

## ETHICAL APPROVAL

Not applicable.

## Data Availability

Data sharing not applicable to this article as no datasets were generated or analyzed during the current study.
